# Neurophysiological biomarkers for Lewy body dementias

**DOI:** 10.1016/j.clinph.2015.06.020

**Published:** 2016-01

**Authors:** Ruth A. Cromarty, Greg J. Elder, Sara Graziadio, Mark Baker, Laura Bonanni, Marco Onofrj, John T. O’Brien, John-Paul Taylor

**Affiliations:** a Institute of Neuroscience, Campus for Aging and Vitality, Newcastle University, Newcastle upon Tyne NE4 5PL, UK; b Institute of Neuroscience, Framlington Place, Newcastle University, Newcastle upon Tyne NE2 4HH, UK; c Clinica Neurologica, Dipartimento di Neuroscienze e Imaging, Università “G.D’Annunzio” Chieti-Pescara, Italy; d Department of Psychiatry, University of Cambridge, Cambridge Biomedical Campus, Cambridge CB2 0SP, UK

**Keywords:** Biomarkers, Dementia with Lewy bodies, Parkinson’s disease with dementia, Cognitive fluctuations, Neurophysiology

## Abstract

**Objective:**

Lewy body dementias (LBD) include both dementia with Lewy bodies (DLB) and Parkinson’s disease with dementia (PDD), and the differentiation of LBD from other neurodegenerative dementias can be difficult. Currently, there are few biomarkers which might assist early diagnosis, map onto LBD symptom severity, and provide metrics of treatment response. Traditionally, biomarkers in LBD have focussed on neuroimaging modalities; however, as biomarkers need to be simple, inexpensive and non-invasive, neurophysiological approaches might also be useful as LBD biomarkers.

**Methods:**

In this review, we searched PubMED and PsycINFO databases in a semi-systematic manner in order to identify potential neurophysiological biomarkers in the LBDs.

**Results:**

We identified 1491 studies; of these, 37 studies specifically examined neurophysiological biomarkers in LBD patients. We found that there was substantial heterogeneity with respect to methodologies and patient cohorts.

**Conclusion:**

Generally, many of the findings have yet to be replicated, although preliminary findings reinforce the potential utility of approaches such as quantitative electroencephalography and motor cortical stimulation paradigms.

**Significance:**

Various neurophysiological techniques have the potential to be useful biomarkers in the LBDs. We recommend that future studies focus on maximising the diagnostic specificity and sensitivity of the most promising neurophysiological biomarkers.

## Highlights

• Biomarkers are needed to improve Lewy body dementia (LBD) diagnosis and measure treatment response.
• There is substantial heterogeneity in neurophysiology biomarker methodologies limiting comparison.
• However, there is tentative evidence to suggest neurophysiological approaches may show promise as potential biomarkers of LBD.

## 1 Introduction

Dementia with Lewy bodies (DLB) is the second most common cause of degenerative dementia in older people after Alzheimer’s disease (AD), where approximately 10–15% of dementia cases demonstrate Lewy body pathology at autopsy (McKeith, 2006). In Parkinson’s disease (PD), dementia is a common outcome and up to 80% of PD patients eventually develop dementia as the disease progresses (Aarsland et al., 2003, Hely et al., 2008). Collectively, DLB and Parkinson’s disease dementia (PDD) can be grouped under the umbrella term of Lewy body dementias (LBD) due to the overlap in symptom profile, similar treatment response, and common underlying neuropathology of alpha-synuclein aggregation (Francis, 2009). Individuals with LBD therefore represent an important disease group in older age, with a significant corresponding impact upon health and society.

Cognitively, LBD patients display marked deficits in executive and visuo-spatial/visuo-perceptual function, and variations in their levels of arousal and attention; the latter are typically referred to as ‘cognitive fluctuations’ (Lee et al., 2012, McKeith et al., 2005, Mollenhauer et al., 2010, Mosimann et al., 2004). Additional clinical features include spontaneous Parkinsonism motor features (McKeith et al., 2005), but non-motor manifestations such as visual hallucinations, autonomic dysfunction, syncope, repeated falls, rapid eye movement sleep behaviour disorder (RBD), delusions and depression are also typical in the LBDs and can cause significant difficulties for patients (McKeith et al., 2005).

There are a number of treatment challenges in the LBDs. Profound cholinergic deficits occur in LBD, and are even more apparent than those observed in AD (Samuel et al., 2000). The remediation of cholinergic function, by the use of cholinesterase inhibitors such as donepezil, rivastigmine and galantamine, may have cognitive and neuropsychiatric benefits, including improvements in global cognitive function, attentional function and activities of daily living (McKeith et al., 2004). However, intra-individual variations are frequently observed in the response to these treatments (Burn and McKeith, 2003) and responder stratification, through the use of apposite biomarkers, would aid the clinical management of LBD. Beyond the cholinesterase inhibitors, there are few efficacious pharmacological treatment options, and agents such as memantine have been tried with mixed success (Aarsland et al., 2009, Emre et al., 2010, Matsunaga et al., 2015). Consequently, there is now a great deal of interest in the search for viable and specific biomarkers in LBD, as these would assist the development of novel therapeutics and provide an accurate method for monitoring treatment response.

Existing candidate biomarkers, which have been used in the LBDs, have included clinical, biochemical, genetic, and neuroimaging markers, such as alpha-synuclein or amyloid beta levels within cerebrospinal fluid (CSF), or alpha-synuclein gene mutations (Hanagasi et al., 2013, Lippa et al., 2007). Generally, the utility of these candidate biomarkers has only been supported in a research context, aside from dopamine transporter imaging, which due to its high specificity in differentiating DLB from AD (McKeith et al., 2007) is now recommended clinically as a method for confirming the diagnosis of DLB in uncertain cases (Mak et al., 2014). However, dopamine transport imaging remains expensive, exposes an individual to radioactivity and provides little or no information regarding the disease progression or prognosis. Additionally, this method does not overtly correlate with the severity of cognitive or neuropsychiatric symptoms in DLB. Similarly whilst magnetic resonance imaging (MRI) has been shown to be a useful tool in the differential diagnosis of the dementias (Mak et al., 2014) its use in LBD is relatively limited and compared to other imaging modalities, MRI has the disadvantage of being relatively high in cost. Alternatively, cerebrospinal fluid (CSF) examination has also been mooted as a potential biomarker in LBD, where one potential application might include the assessment of alpha-synuclein levels (Mukaetova-Ladinska et al., 2010). However, the clinical utility of this approach remains uncertain, and further methodological developments are required prior to routine use (Lim et al., 2013). A further disadvantage of the use of CSF as a biomarker is that it cannot be collected in a non-invasive manner.

In summary, there is a clear and pressing need to identify useful biomarkers of LBD in order to: (1) expedite the early diagnosis of LBD and enable differential diagnosis to be obtained, particularly during the prodromal phase of the disease; (2) improve our understanding of LBD progression; (3) provide a means to accurately monitor the therapeutic response to treatment; and (4) ultimately develop early disease-modifying interventions. One modality which has not been extensively examined in LBD is the use of neurophysiological approaches, despite their increasing relevance in AD (de Waal et al., 2011, van Straaten et al., 2014). As biomarkers should ideally be non-invasive, inexpensive, simple to use and technically validated (Gerlach et al., 2012), in this regard, a variety of neurophysiological techniques, and in particular electroencephalography (EEG), may be useful biomarkers of LBD.

In the present review we therefore sought to explore the relevant literature in order to identify the way in which neurophysiological approaches have been applied in LBD. Specifically, we sought to evaluate their diagnostic utility, assess whether these markers map onto symptomatic phenotypes, and finally, examine their performance as potential markers of treatment response.

## 2 Method

### 2.1 Search methods and inclusion/exclusion criteria

In order to identify the available literature regarding current and potential neurophysiological biomarkers in LBD, PubMED (until 29 April 2015) and PsycINFO (from 1967 until April Week 3 2015) databases were searched independently by two of the authors (RAC & GJE) using the following terms: “Lewy^∗^”, “Parkinson’s disease with dementia”, “Parkinson’s disease with mild cognitive impairment”, “dementia with Lewy^∗^” AND “bereitschaftspotential”, “biomarker”, “blink recovery”, “blink reflex”, “contingent negative variation”, “cortical silent periods”, “EEG”, “electroencephalography”, “electrophysiology”, “ERP”, “event-related potential”, “evoked potential”, “flicker fusion”, “flutter fusion” “H-reflex”, “induced potential”, “intra-cortical facilitation”, “ipsilateral silent periods”, “LDAEP”, “long-interval intracortical inhibition”, “long-latency stretch reflex”, “magnetoencephalography”, “MEG”, “mismatch negativity”, “motor evoked potential”, “nerve stimulation”, “neurophysiology”, “prepulse inhibition”, “SAI”, “short afferent inhibition”, “short-interval intracortical inhibition”, “startle”, “sympathetic skin response”, “TMS” and “transcranial magnetic stimulation”.

**Fig. 1:**
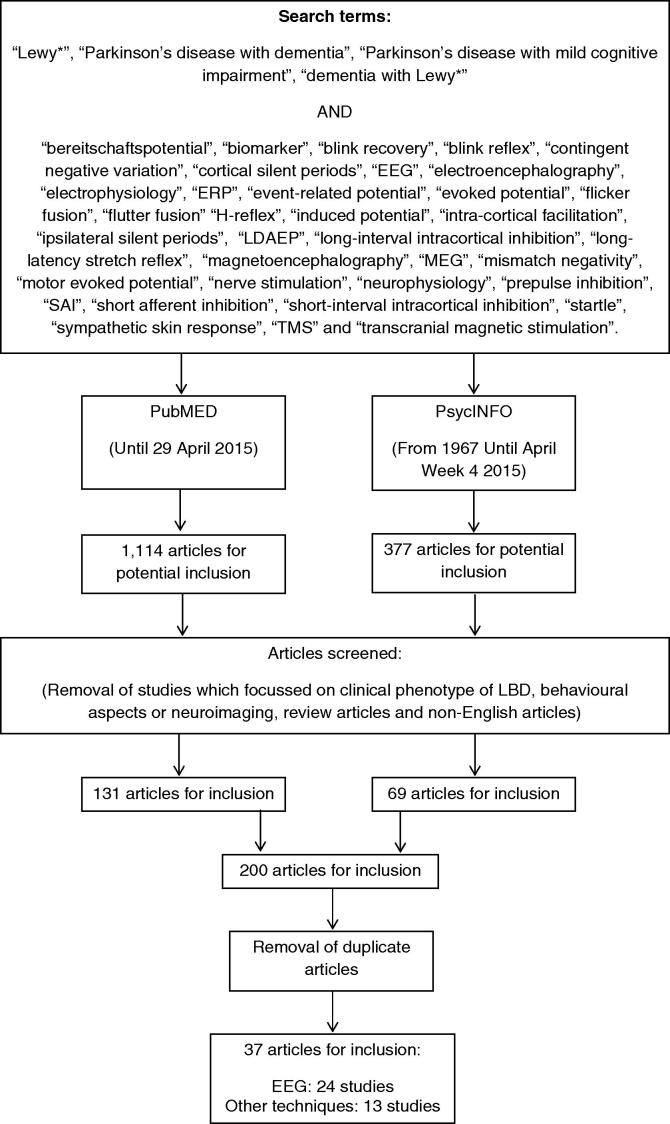
Search strategy and selection criteria.

Studies which focussed only on the clinical phenotype of LBD, those which focussed on the behavioural aspects of LBD, or studies which employed exclusively neuroimaging techniques, were excluded. This search strategy (Fig. 1) resulted in a total of 1491 potential articles. Article titles and abstracts were screened for relevance, and the reference sections of included papers were searched in order to identify any additional studies. Review, non-English and duplicate articles were removed.

For an article to be included, LBD participants were required to have met established diagnostic criteria for either probable DLB or PDD (Emre et al., 2007, McKeith et al., 2005), or, for PDD studies published prior to the 2007 criteria, on the basis of the fourth edition of the Diagnostic and Statistical Manual of Mental Disorders (DSM-IV) (American Psychiatric Association, 1994), or LBD participants who had neuropathological post-mortem confirmation of their diagnosis, in accordance with previously-published guidelines (Fujishiro et al., 2008, McKeith et al., 2005). This resulted in a final total of 37 studies.

## 3 Results

The most common modality used was EEG, as a total of 24 EEG studies were identified (see Supplementary Table S1 for details); 15 of which predominantly examined resting-state EEG and 6 of which examined event-related potentials (ERPs). A total of 12 studies employed a range of other neurophysiological techniques (see Supplementary Table S2) including TMS (5 studies), MEG (2 studies), and the assessment of the blink reflex (2 studies). Studies which examined autonomic features, including skin response and heart rate variability (2 studies), and one study which assessed the auditory startle reaction were also considered. Due to the heterogeneity of studies and the wide range of neurophysiological approaches, these data were not amenable to meta-analysis. Therefore, only narrative descriptions of these studies are provided. An overview of the neurophysiological biomarkers examined and their presumed functional significance in LBD are included in Table 1.

## 4 Discussion

### 4.1 Electroencephalography

EEG recordings can be obtained during the presentation of a particular stimulus, or in the absence of a stimulus; typically collected with the patient’s eyes closed (i.e. “resting-state” EEG). For both approaches, the resulting activity can be analysed in various ways. Event-related activity can be analysed in the time domain by examining event-related potentials (ERPs); referring to positive or negative waveforms (i.e. deflections in the signal) which occur following the presentation of a stimulus. For example, the latency or amplitude of the resulting ERP can be analysed. Alternatively, frequency bands (where the most commonly-investigated bands are delta, theta, alpha, beta and gamma) can be examined, in order to investigate, for example, the observed power within a frequency band, which can then be compared between patient groups and healthy control individuals.

Various EEG analytical techniques have been employed in LBD, such as the use of the Grand Total EEG (GTE) score (a graded composite measure which takes the presence of EEG features such as diffuse slow-wave activity or focal abnormalities into account), the analysis of frontal intermittent rhythmic delta activity, and the use of compressed spectral arrays (Bonanni et al., 2008; Lee et al., 2015, Roks et al., 2008). A long-standing EEG feature of DLB is the presence of posterior (temporal) transient slow or sharp waves (Barber et al., 2000, Briel et al., 1999); the diagnostic criteria for DLB includes these as a supportive feature of the condition (McKeith et al., 2005). More recent work has noted that EEG abnormalities are typically observed in posterior regions in DLB, whereas AD patients generally exhibit EEG abnormalities in temporal areas (Bonanni et al., 2010, Bonanni et al., 2008), suggesting that topographical EEG differences are apparent between DLB and AD.

More broadly, LBD patients typically exhibit increased posterior slow-wave activity relative to healthy controls and individuals with AD (Bonanni et al., 2008, Briel et al., 1999, Calzetti et al., 2002, Fernandez-Torre et al., 2007, Roks et al., 2008). Clinically, this increased slow-wave activity has been found to positively correlate with the presence and severity of cognitive fluctuations (Bonanni et al., 2008, Walker et al., 2000b). Alterations to EEG activity also appear to associate with cognition in the LBDs. Schlede and colleagues investigated an overall measure of EEG alteration known as the ‘Short Grand Total EEG (GTE)’ score in LBD, where the Short GTE score was comprised of six separate subscores calculated by visually interpreting certain EEG features (e.g. diffuse slow activity). The study showed that Short GTE scores, where higher Short GTE scores indicated more impairment, negatively correlated with a combined MMSE and Clock Drawing Task (CDT) composite score (Schlede et al., 2011).

One EEG study demonstrated that DLB patients, who were not taking donepezil, showed increased spectral power density in the delta and theta bands compared to a control group, which was not evident in individuals with AD when similarly compared to the control group (Kai et al., 2005). A greater degree of parietal delta power band variability has also been reported in patients with DLB, relative to AD patients and controls (Andersson et al., 2008). However, increased power in these bands in individuals with AD has been reported in at least four studies (Jackson and Snyder, 2008), and thus may represent a non-specific ‘dementia’ characteristic. Further comparisons between LBD and AD are therefore warranted to determine if there is a spectral power density differential between the two groups, which is sufficient to be useful diagnostically.

Measures of EEG coherence have also been examined, which is a measure indicative of functional cortical connectivity and in the context of LBD, a measure which may highlight modulatory effects of cholinergic deficits (Adler et al., 2003). One such study examined EEG coherence between four regions (left anterior, right anterior, left posterior and right posterior) in DLB patients. Compared to controls, DLB patients showed greater extended coherence (average coherence between all regions) in the delta band, but reduced extended coherence in the alpha band (Andersson et al., 2008). In a similar study, Kai and colleagues also observed differences in DLB and AD patients in terms of intrahemispheric coherence values, and differences between DLB and AD in fronto-temporocentral delta and theta intrahemispheric coherence values, and temporo-centro-parieto-occipital beta band coherence values; a result which the authors speculated was due to the greater cholinergic dysfunction in DLB (Kai et al., 2005).

In another study, Caviness and colleagues analysed resting EEG from PD, PD-MCI and PDD patient groups and observed that the mean dominant posterior background rhythm frequency (DPBRF) was significantly different between all patient groups (Caviness et al., 2007). The lowest DPBRF was observed in PDD patients (7.1 Hz) compared to PD-MCI and PD groups (8.1 Hz and 9.1 Hz respectively). The groups also differed in terms of delta activity, where significant differences were shown between PDD and PD-MCI groups. There were also variations in both delta and theta bands in PDD patients compared to individuals with PD, where PDD patients showed an increased percentage of global relative power in both bands. However, as this study did not correct for multiple comparisons and included a limited number of PDD patients (*n = *8), these results should be interpreted with caution.

The greater temporal resolution afforded by EEG compared to, for example, neuroimaging methods such as functional blood oxygen level dependent (BOLD) functional magnetic resonance imaging (fMRI), may offer better aetiological perspectives into the fluctuating perturbations in brain networks that occur in neurodegenerative disease. Certainly in EEG recordings, DLB patients have also displayed greater fluctuations in frequencies on a second-by-second basis compared to AD patients and controls, and these changes in EEG have been causally associated with the severity and frequency of cognitive fluctuations (Bonanni et al., 2008, Walker et al., 2000a). Moreover, the frequency variability in posterior regions also appears to be reduced by the administration of cholinesterase inhibitors (Kai et al., 2005), suggesting that EEG parameters could be used as a neurophysiological biomarker of treatment response. Work conducted in patients with AD has shown that cholinesterase inhibitors can affect resting-state EEG activity, where reductions in delta or theta activity have been observed after successful treatment, accompanied by increases in dominant alpha activity and cognitive functioning (Babiloni et al., 2013). It has been suggested that in AD, the use of EEG neurophysiological biomarkers might aid the development of new therapeutic treatments, particularly when used alongside other imaging, biological or cognitive markers (Babiloni et al., 2013) and thus by extension the use of EEG might also be useful in the LBDs.

One other potential use of EEG which merits further attention is in the identification of individuals in the prodromal phase of the LBDs, prior to the manifestation of significant cognitive impairment. From a theoretical perspective EEG, as a functional marker of neuronal and synaptic integrity, may be sensitive to subtle and early neurodegenerative changes that precede overt large-scale neurodegenerative structural changes in LBD. Empirically, as noted above, Caviness and colleagues (2007) observed that global delta activity was intermediate in PD-MCI patients, compared to individuals with PD and PDD. One additional study noted that on the basis of resting-state EEG recorded from 17 individuals with PD-MCI and nine with PDD, PDD patients showed significantly increased beta peak frequency, and decreased alpha relative power and alpha/theta power, compared to PD-MCI patients at one and two-year follow-ups (Gu et al., in press). A larger longitudinal study followed-up 47 individuals with mild cognitive impairment (MCI), 50 individuals with DLB, 50 with AD and 50 healthy controls for a period of three years (Bonanni et al., 2015). At the end of the three-year period, 20 individuals with MCI converted to probable DLB, and notably, 100% of these individuals displayed EEG abnormalities which are typical of DLB at baseline, including a reduced dominant frequency and increased dominant frequency variability. Despite the potential limitations of this study, including the focus on individuals with MCI who were referred to a tertiary centre and a lack of validation in independent cohorts, these data intriguingly suggest that EEG might be a useful neurophysiological biomarker for patients who are on a trajectory towards DLB.

Technical aspects of EEG are likely to impact upon clinical studies in LBD, and these are likely to be affected by the classic limitation of EEG, which is the ‘inverse problem’, which refers to the difficulties in using spatial EEG measurements to confirm the actual source of the EEG signal (Koles, 1998). Although various different source localisation methods have been employed to address this, there is no accepted gold-standard analytical method (Michel et al., 2004). This potentially limits the utility of EEG, based on the topographical findings to date, in addressing aetiological questions in LBD. Similarly, the ability of the EEG to directly measure deep cortical and subcortical activity is poor; nevertheless, subcortical areas and the basal ganglia are likely to be involved in the pathophysiology of LBD (Peraza et al., 2014). Thus, alternative methods to EEG are needed to probe these structures and understand their involvement in LBD symptoms. For example, it would be useful to confirm whether posterior cortical areas are implicated in the occurrence of cognitive fluctuations, or alternatively if these symptom phenomena arise from more diffuse cortical, or indeed subcortical and thalamocortical, neural perturbations.

Several EEG studies with negative findings have been observed in the LBDs. In one study, resting EEG recordings obtained from a longitudinal dementia study were retrospectively analysed; clinical diagnoses of AD (*n = *45) or DLB (*n = *48) were also made retrospectively in this study (Londos et al., 2003). The EEG recordings indicated that theta activity was dominant in both groups with generalised slow-wave activity, but that there were no significant differences between AD and DLB. However, this study relied on visual inspection of the EEG rather than objective quantification methods. Whilst subjective approaches to the analysis of EEG data might provide useful information regarding the underlying pathophysiology, and determine the presence of DLB or AD compared to control patients (Liedorp et al., 2009), the major limitation of these visual inspection approaches is their poor specificity, despite the good levels of sensitivity (Brenner et al., 1988, Hooijer et al., 1990).

Advanced semi-automated or fully automated statistical quantitative EEG methods, which consider a range of EEG temporal and spatial features, have allowed for marked improvements in specificity in separating DLB from AD (Bonanni et al., 2010, Bonanni et al., 2015, Bonanni et al., 2008, Snaedal et al., 2012). Such approaches have included the application of compressed spectral arrays (Bonanni et al., 2010, Bonanni et al., 2015, Bonanni et al., 2008), a technique developed for EEG monitoring during coma (Karnaze et al., 1982), which can be easily used in a clinical environment and displays the power expressed by frequency bands every two seconds. This provides a rapid on-line approach to assessing EEG frequency variability, which is a key feature in LBD. Others have applied statistical pattern recognition algorithms in order to discriminate between different dementia subtypes (Engedal et al., 2015, Snaedal et al., 2012). Overall, whilst these techniques are promising, their widespread translation to clinical practice has not yet occurred and these approaches need to be replicated and validated outside of their respective research centres.

### 4.2 Event related potential studies

When a particular stimulus (e.g. auditory or visual) is presented to an individual with concurrent EEG monitoring, event-related potentials (ERPs) can be observed in the subsequent time-locked EEG activity. ERPs are defined in accordance with the polarity, referring to whether the resulting waveform is positive or negative, and in terms of the latency following the stimulus presentation, which is measured in milliseconds (ms). For example, the ‘P300’ ERP represents a positive waveform, observed 300 ms following the presentation of a stimulus (Luck, 2005). ERP studies thus might offer a method by which attentional or other cognitive deficits in LBD can be accurately characterised.

Although the current literature on ERPs in LBD patients is relatively sparse (see Supplementary Table S1), several studies have demonstrated that atypical ERPs are a common feature of LBD (Bonanni et al., 2010, Kurita et al., 2010, Perriol et al., 2005, Pugnetti et al., 2010). Whilst the use of ERPs in the LBDs may have important implications for understanding pathophysiology; for example, by providing a better understanding of attentional dysfunction or visual hallucinations (Brønnick et al., 2010, Kurita et al., 2010), there is less evidence to suggest that they are useful from a diagnostic perspective, as generally, these studies do not report sensitivities and specificities.

One study conducted by Bonanni and colleagues, using an auditory ‘oddball’ paradigm, showed that the P300 was lower in amplitude and had a greater latency in DLB patients compared to AD patients (Bonanni et al., 2010), and the pattern of P300 amplitude gradients was inverted (i.e. there was a higher amplitude in frontal leads and smaller amplitude in posterior leads in DLB patients compared to AD and controls). This may appear counterintuitive, as reduced amplitudes might be expected in LBD, which is characterised by early frontal lobe involvement with the co-occurrence of dysexecutive symptoms (Dodel et al., 2008). Nevertheless, delayed P300 latencies were evident in DLB patients studied in anterior, rather than posterior, regions compared to AD, which is compatible with the cognitive phenotype common to LBD. Furthermore, the delayed P300 response in DLB correlates with the severity of cognitive fluctuations, thus suggesting that the P300 latency may also serve as a useful objective neurophysiological biomarker for the severity of this core symptom (Bonanni et al., 2010). From a clinical perspective, the presence of a reversed amplitude distribution, in those with a reliable P300 response, differentiated DLB from AD patients with a sensitivity of 70% and specificity of 97% (Bonanni et al., 2010).

Other approaches have included the use of the prepulse inhibition (PPI) paradigms in conjunction with simultaneous EEG recording; to date, only one study has employed this method in patients with LBD (Perriol et al., 2005). The PPI paradigm involves the presentation of a weak auditory stimulus immediately prior to the presentation of a stronger stimulus, resulting in a ‘startle response’. Typically, in healthy individuals the second pulse is perceived as irrelevant and is filtered out, which is reflected in suppression of the ERP waveform (Oranje et al., 2006). Perriol and colleagues investigated attentional selectivity using the PPI paradigm in AD, DLB, PDD and healthy control individuals, where an 80 decibel (dB) sound pulse preceded a 115 dB sound pulse (Perriol et al., 2005). The percent PPI of the N100/P200 component amplitude was significantly reduced in the DLB group relative to AD patients and controls, whilst the PDD group exhibited intermediate PPI disturbances, where the percentage of PPI was significantly reduced compared to controls. The authors speculated that the reduced PPI reflects a more severe disruption of the dopaminergic subcortico-thalamo-cortical networks in DLB compared to PDD (Perriol et al., 2005).

From the limited work thus far, it is evident that ERP studies may help make significant inroads into understanding clinical symptoms and cognitive dysfunction in LBD, as well as potentially providing ways to differentiate LBD from other dementias. However, aside from technical issues such as the inverse problem, other challenges remain. Due to the nature of ERP-associated tasks, it is necessary that an individual has the ability to sustain their attention to task stimuli over a large number of trials in order to generate an adequate signal-to-noise ratio. Effectively completing a paradigm may be particularly difficult for LBD patients who have significant cognitive impairment and/or who experience fluctuations in attention.

### 4.3 Magnetoencephalography

Magnetoencephalography (MEG) provides another ‘window’ into the brain and involves the detection of the electromagnetic field generated by postsynaptic currents in cortical pyramidal neurons (Hari and Salmelin, 2012). Compared to EEG, because the magnetic field is not attenuated by the skull or scalp, MEG can be used to acquire very high-frequency activity which cannot be captured using EEG (Hari et al., 2010). MEG is sensitive to cortical currents which are tangential to the skull and in cortical fissure walls, whereas EEG detects currents in all orientations (Hari et al., 2010, Hari and Salmelin, 2012). Importantly, MEG does not require a reference source (i.e. where the signal is compared to a signal measured from a separate location) and can also measure neural activity without requiring physical contact with the skull, which can increase the speed of application (Hämäläinen et al., 1993, Hari et al., 2010).

Two resting-state MEG studies have reported abnormal cortical rhythms in LBD patients with evidence of cortical rhythm slowing, particularly in posterior regions, consistent with EEG data (Bosboom et al., 2009, Franciotti et al., 2006). In the first study, Franciotti and colleagues analysed a range of frequency bands in a small number of moderate and severe AD and LBD patients, and control individuals, during conditions in which participants had their eyes closed or eyes open. EEG recordings were also obtained during a simple mental arithmetic task. The measurement of alpha and pre-alpha cortical reactivity, during both open and eyes-closed conditions, was able to differentiate control participants from patients, and also separated individuals with moderate AD from those with severe AD and LBD (Franciotti et al., 2006). However, it is unclear if this study indicates that the underlying activity observed in patients with severe AD is similar to the underlying activity in DLB.

In the second study, Bosboom and colleagues conducted a resting-state MEG study during which they treated a small sample of eight PDD patients with the cholinesterase inhibitor rivastigmine (Bosboom et al., 2009). The treatment was found to reverse some of the slowing of resting-state activity (resulting in a shift in the frequency spectrum towards higher frequencies), providing further support for the notion of cholinergic system dysregulation in PDD. Interestingly, greater levels of post-treatment cognitive improvements were positively associated with a smaller reduction in relative delta power, suggesting that a better clinical response was associated with lower baseline levels of delta power. A negative association was also found between cognitive improvements and relative theta power changes. These data potentially indicate that the mechanism by which rivastigmine exerts therapeutic effects in LBD patients, particularly the amelioration of cognitive deficits (Maclean et al., 2001), could be due to the normalisation of the cortical rhythms (i.e. a shift towards higher frequencies). Speculatively, cortical rhythms observed during resting-state MEG, and post-treatment changes in the frequency of resting-state cortical rhythms, might be effective neurophysiological biomarkers of cholinesterase inhibitor (or other agents) treatment efficacy in LBD. However, no further MEG studies have been conducted to date in order to validate this. From a clinical perspective, the use of MEG in the diagnosis and monitoring of LBD is, however, likely to be limited given the relative expense of MEG facilities and lack of availability, as they are often located only in major research centres.

### 4.4 Blink reflex studies

The blink reflex is typically used to assess lesions to the brainstem and involves the measurement of the reaction to electrical stimulation of the supraorbital nerve, from which the latency to three separate responses can be derived (Bonanni et al., 2007). The first response, R1, is generated in the trigemino-facial reflex arc, and R2 and R3 are generated in polysynaptic pathways involving the brainstem reticular formation (Bonanni et al., 2007).

Bonanni and colleagues hypothesised that as brainstem neuropathology has been proposed to be a feature of early LBDs (Braak et al., 2003), the blink reflex might be abnormal in DLB and particularly in patients with rapid eye movement sleep behaviour disorder (RBD) (Bonanni et al., 2007). In their study, healthy controls and patients with DLB, multiple system atrophy (MSA), progressive supranuclear palsy, and AD were assessed. The DLB group showed a significantly delayed blink reflex response (increased R2 latency) relative to all other groups (Bonanni et al., 2007). These results were independent of the presence or absence of RBD, as delayed R2 latencies were observed in five DLB patients who did not display RBD, suggesting that R2 latencies are not a marker of brainstem neuropathology. Whilst the R2 latency might be a useful diagnostic biomarker, to date, no other blink reflex studies have been carried out in order to confirm whether this neurophysiological marker is sufficiently robust to diagnostically separate DLB from other patient groups.

From a treatment response perspective, the blink reflex may have utility as a biomarker; Anzellotti and colleagues investigated the effect of the cholinesterase inhibitor donepezil upon the blink reflex response in 26 DLB patients and 20 AD patients (Anzellotti et al., 2008). Blink reflexes were assessed at a baseline period and following treatment with donepezil. In the DLB group, the R2 latency of the blink reflex was significantly reduced by 8.2% relative to baseline and was accompanied by a post-treatment reduction in cognitive fluctuations. However, the blink reflex response does suffer from some limitations which may inhibit its clinical use; firstly, blink reflex recording is technically difficult, as the electrical pulse delivered to the supraorbital trigeminal nerve branch can spread and distort the subsequent measurement and secondly, the stimulus can be unpleasant or moderately painful.

### 4.5 Other techniques

Aside from the methodologies mentioned above, a variety of other neurophysiological techniques have been employed in order to investigate LBD. Kofler and colleagues investigated the auditory startle reaction (ASR) in patients with DLB compared to patients with other Parkinsonian disorders and healthy control individuals (Kofler et al., 2001). The ASR paradigm in this study involved presenting participants, who reclined on a bed in a quiet semi-darkened room, with auditory tone bursts of a random frequency and intensity. The subsequent effects upon EMG measurements, obtained from a range of areas (orbicularis oculi, masseter, sternocleidomastoid, biceps brachii, abductor pollicis brevis, rectus femoris, tibialis anterior, and soleus muscles), were then examined. DLB patients showed an abnormal ASR profile, displaying fewer ASRs than healthy control individuals and virtually no responses in lower extremities. The probability of ASRs was similar in facial areas, the neck and in the upper extremities when DLB patients were compared to patients with PD or with MSA, although the amplitude of ASRs were markedly lower in DLB patients compared to PD patients and control individuals, which the authors speculated may have been as a result of brainstem pathology occurring in DLB (Kofler et al., 2001).

Alternative physiological biomarkers might include the examination of autonomic abnormalities. Negami and colleagues examined the sympathetic skin response (SSR) and heart rate variability (HRV) in a comparison of patients with AD (*n = *20) with a group of DLB patients (*n = *20) (Negami et al., 2013). The SSR, which reflects the sympathetic sweat response and which was measured by the response to induced median nerve stimulation, was shown to be lower in individuals with DLB compared to AD. Additionally, the ratio of low-frequency to high-frequency power in HRV was significantly lower in DLB patients compared to AD. Based on the cut-off values employed in this study, the sensitivities of the SSR and HRV were 85% and 90% respectively, and the specificity of each technique was 85% and 85%. Similarly, Akaogi and colleagues also observed autonomic differences in DLB and PDD patients relative to PD patients and controls (Akaogi et al., 2009). In this study, the sympathetic sweat response and skin vasomotor reflex were measured from the palm of the hand. Cardiovascular function was also assessed, expressed as the coefficient of variation of R-R intervals (CV_R–R_) measured in response to the head-up tilt test. The LBD and PD patients displayed reduced sweat response amplitudes compared to controls, and the vasomotor reflex amplitudes were also lower in LBD, but not PD patients, compared to controls. In addition, DLB patients showed a significantly lower CV_R–R_ value (Akaogi et al., 2009).

Overall these findings suggest that autonomic biomarkers merit further investigation in LBDs. They may also be particularly salient to the detection of early disease (i.e. prodromal DLB) given the expectation that autonomic symptoms may predate the manifestation of cognitive and neuropsychiatric symptoms in Lewy body disease by a number of years (Donaghy et al., 2015).

### 4.6 Transcranial magnetic stimulation

**Fig. 2:**
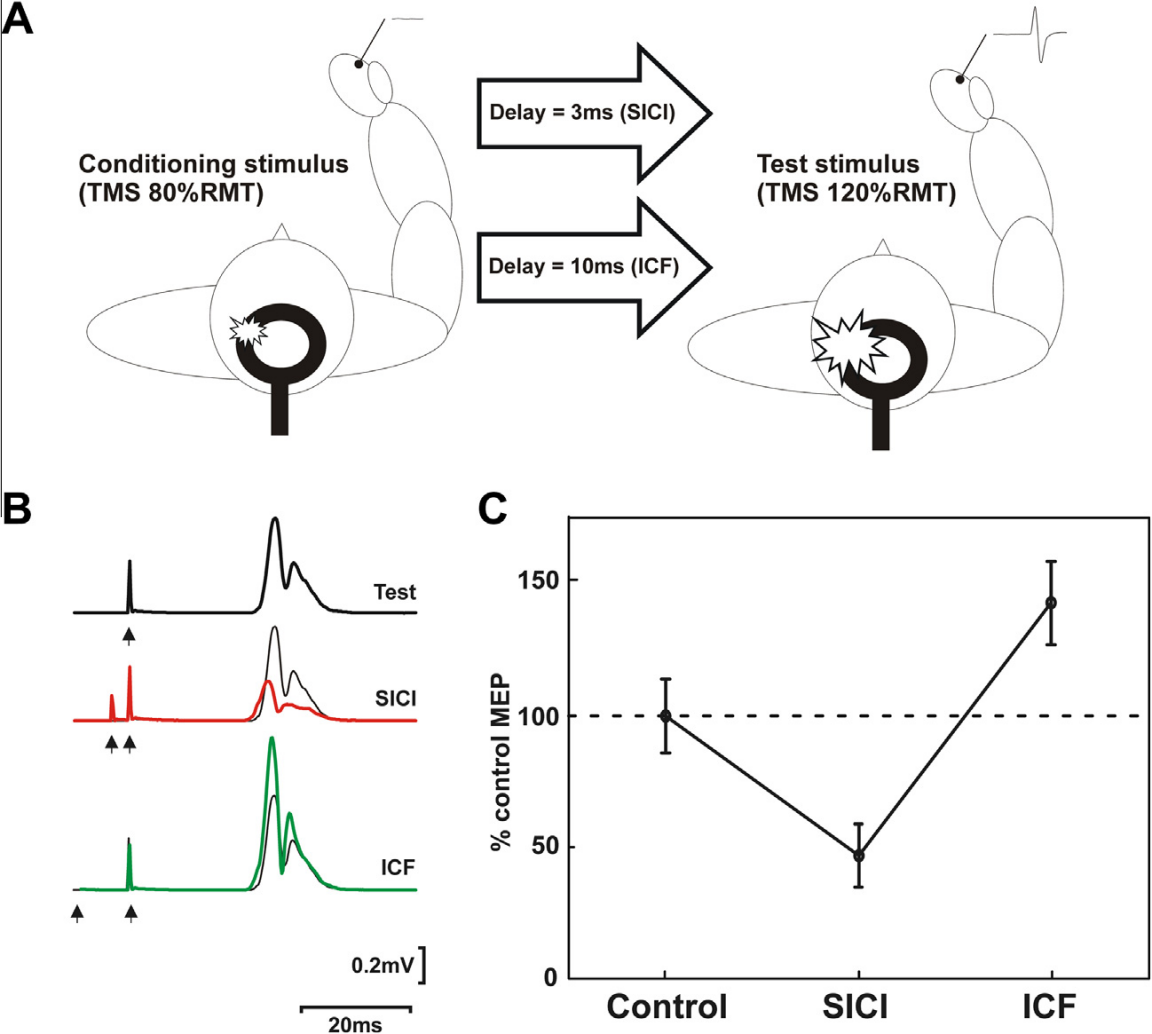
Short-interval intracortical inhibition (SICI) and intracortical facilitation (ICF). (A) Figure showing the experimental procedure for SICI/ICF. (B) Conditioned and unconditioned MEPs from a 50 year old healthy control participant at rest (i.e. no background EMG contraction). To measure both SICI and intracortical facilitation (ICF) TMS conditioning pulses were delivered at 80% resting motor threshold (RMT) and test pulses were delivered at 120% RMT. Unconditioned MEPs are plotted in black and conditioned MEPs are plotted either in red (3 ms interstimulus interval; SICI), or green (10 ms interstimulus interval; ICF). Each trace is an average of 20 individual rectified MEPs. (C) Comparison of mean SICI and ICF in healthy control participant. Each data point is an average of 20 MEPs. Test MEPs are normalised to the unconditioned (control) MEP. Error bars represent standard error. In the age-matched control MEPs are significantly affected by short interval and long interval conditioning (two-tailed *t* test; *p* < 0.05). (For interpretation of the references to color in this figure legend, the reader is referred to the web version of this article.)

**Fig. 3:**
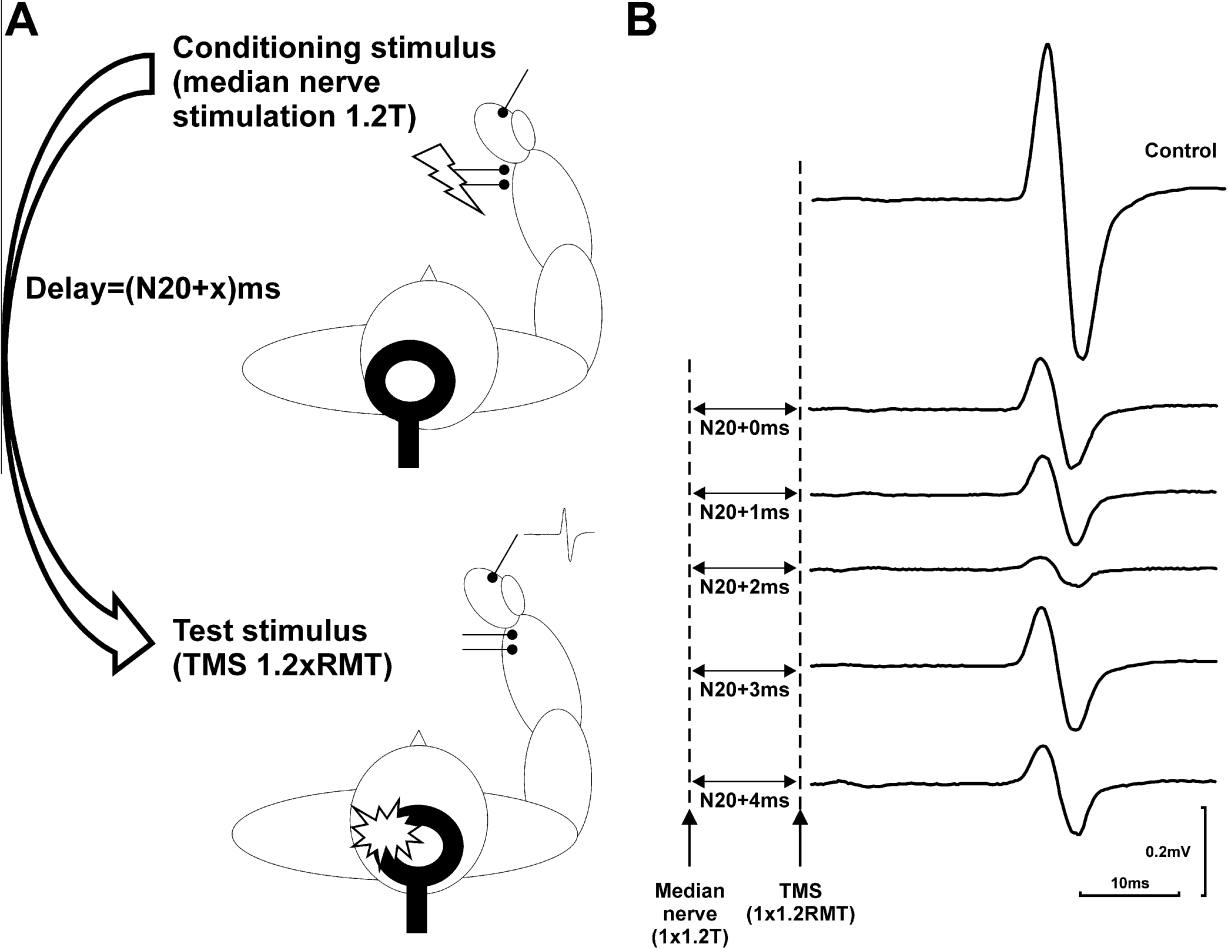
Short-latency afferent inhibition (SAI). (A) Experimental procedure for SAI. (B) Examples of conditioned and unconditioned motor evoked potentials (MEPs) produced by a short-latency afferent inhibition (SAI) protocol in a 66-year-old control participant. Time intervals between the conditioning median nerve stimulation (set 20% above threshold for observing a twitch in abductor pollicis brevis) and the TMS test pulse (set at 1.2 times resting motor threshold) are expressed relative to the latency of the N20 component of the somatosensory evoked potential (SEP) measured in each individual (e.g. N20+2 ms). Averaged unrectified MEPs have been plotted (each trace represents an average of 20 individual sweeps). Time voltage calibration bars apply to all MEPs, but note the colour coding for voltage calibration).

Transcranial magnetic stimulation (TMS) is a non-invasive cortical stimulation technique which has been used in LBD in order to evaluate excitatory/inhibitory characteristics of the cortex, and in particular, the corticomotor system. Examples of TMS approaches include short intra-cortical inhibition (SICI; Figs. 2A and C), intra-cortical facilitation (ICF; Figs. 2B and C) and short-latency afferent inhibition (SAI; Fig. 3A).

To date, the application of these cortico-motor parameters to LBD cohorts has been limited. Two studies have examined SICI, a cortico-motor based measure which is a possible surrogate for GABAergic function (Stagg et al., 2011), in DLB (Di Lazzaro et al., 2007, Nardone et al., 2006); however, in the study conducted by Di Lazzaro and colleagues, no significant differences were observed in SICI between DLB, AD and controls (Di Lazzaro et al., 2007). Similarly, Nardone and colleagues reported no significant differences in SICI in DLB patients compared to controls, although reductions were shown in AD patients (Nardone et al., 2006). Work in PD cohorts has suggested that alterations to cortical silent periods (cSPs) and ipsilateral silent periods (iSPs) might be a feature of PD (Morita et al., 2008), but it is not known whether changes in these cortico-motor parameters extend into the LBDs. Other TMS methodologies include the measurement of motor evoked potential (MEP) thresholds, long-interval intracortical inhibition (LICI) (Cantone et al., 2014) and ‘paired-pulse’ protocols, which can measure cortical interhemispheric inhibition and transcallosal function (Ferbert et al., 1992). However, studies are yet to examine these measures in LBD.

One cortico-motor TMS parameter which has been applied more extensively in DLB cohorts is that of short-latency afferent inhibition (Fig. 3) (Celebi et al., 2012, Marra et al., 2012, Nardone et al., 2006). This measure has been mooted to be an *in vivo* biomarker of cholinergic function and has been reported to be reduced in dementias which have significant neuropathologic evidence of cholinergic loss. Therefore, for example, SAI alterations are not observed in fronto-temporal dementia (Di Lazzaro et al., 2006) but DLB patients show a reduced SAI compared to healthy controls (Di Lazzaro et al., 2007, Marra et al., 2012). However, the SAI literature is not consistent in DLB; for example, Nardone and colleagues did not observe any SAI or SICI differences between DLB patients and controls (Nardone et al., 2006).

From a PDD perspective, despite numerous reports on SAI in PD (Nardone et al., 2005, Rochester et al., 2012, Sailer et al., 2003, Yarnall et al., 2013), only one preliminary report has demonstrated that SAI is reduced in PDD compared with PD (Celebi et al., 2012). Compared to cognitively normal patients with PD and healthy controls, individuals with PD-MCI have been shown to have a significantly reduced SAI, indicating that SAI could be a useful neurophysiological biomarker of cholinergic dysfunction, even in the earliest stages of cognitive decline (Yarnall et al., 2013). However, comparable data in DLB are currently lacking.

SAI, as an indirect *in vivo* measure of cholinergic function, might also serve as a means of understanding symptom aetiology in LBD; a small number of reports have examined the relationship between SAI function and cognitive and neuropsychiatric symptoms in LBD. One study found that SAI was reduced in non-demented PD patients with visual hallucinations compared to those without, and that these changes were accompanied by selective deficits in attentional and visuospatial function (Manganelli et al., 2009). Therefore, a reduction in SAI, in the presence of visual hallucinations and subtle cognitive impairment, may be related to cholinergic dysfunction in PD patients at a higher risk of progressing to dementia. Related to this, SAI also appears to correlate with visual hallucination severity in DLB, further supporting the argument that cholinergic deficits are apposite to the aetiology of this core symptom (Marra et al., 2012). However, relevant factors including disease heterogeneity, small sample sizes, and concurrent psychotropic medication use, which is known to influence the effects of TMS (Ziemann, 2004), might limit the clinical utility of SAI as a neurophysiological biomarker in the LBDs, as these factors have undoubtedly contributed to the variable SAI findings observed in LBD (Di Lazzaro et al., 2005, Nardone et al., 2005).

Outside of the corticomotor system, TMS has been applied to the visual cortex to help address the aetiology of visual hallucinations in DLB. Phosphene thresholds, which provide a marker of cortico visual excitability, were evaluated in DLB patients compared to controls in a study by Taylor and colleagues (Taylor et al., 2011). This study showed that phosphene thresholds and their associated parameters were not significantly different between groups, but there was a positive correlation between visual cortical hyperexcitability and the severity and frequency of visual hallucinations. The authors therefore suggested that whilst increases in visual cortical excitability do not explain visual hallucinations, TMS visual excitability might be a neurophysiological biomarker of symptom frequency and severity. Whilst TMS is undoubtedly a promising research technique, it may be unsuitable for routine clinical use due to the time-consuming and technical nature of TMS protocol delivery.

## 5 Conclusions

It is evident that a wide range of neurophysiological approaches can potentially be used as useful LBD biomarkers. Potential uses include: the accurate and reliable identification of individuals with LBD compared to other dementias or healthy individuals; as objective markers of cognitive or neuropsychiatric symptom severity, or, as a marker of treatment response.

However, there are a series of limitations inherent in the studies conducted to date. Firstly, of the studies which have examined neurophysiological biomarkers in the LBDs comparing patient groups, sample sizes have been relatively small (e.g.; Briel et al., 1999, Calzetti et al., 2002, Kai et al., 2005, Schlede et al., 2011). In addition to the small sample size issue, even when patients included in the studies have met the relevant diagnostic criteria for DLB and PDD, there has been a great deal of heterogeneity in the patient cohorts. This is particularly apparent with regard to the disease severity and underlying disease pathology.

The use of medication in the studies reported to date is also a potential limitation. Cholinesterase inhibitors are generally used in patients with DLB, and levodopa medications are generally used in patients with significant parkinsonian symptoms; however, little is known regarding the effects of these medications, or combinations thereof, upon the range of neurophysiological biomarkers discussed in the present review. The evidence from the studies included in the review is mixed, as whilst the use of medication (anti-depressants, benzodiazepines and neuroleptics) was not shown to affect measures of EEG coherence in one study (Andersson et al., 2008), the administration of donepezil was shown to affect delta and theta band mean power in another EEG study (Kai et al., 2005).

Another limitation is that the replication of previous studies, in larger cohorts, is lacking. Such studies are necessary in order to accurately assess the diagnostic utility of the neurophysiological markers in LBD. Furthermore, not all studies have reported levels of specificity and sensitivity when using neurophysiological markers to compare between clinical groups of patients, and where cut-off values have been used to separate groups of individuals with different types of dementia, it has generally been the case that the cut-off values employed have not been specified in advance (e.g. Andersson et al., 2008). These challenges undermine the possibility of carrying out robust meta-analytic testing of neurophysiological biomarkers in LBD.

In order to overcome these clear methodological limitations and increase the utility of neurophysiological biomarkers, we recommend the following, as a research priority:

(1) Replicative studies, with large numbers of patients, are needed. This will allow the accurate assessment of the validity and reliability of neurophysiological markers and enable meta-analyses to be conducted.
(2) The effects of common pharmacological agents used as treatment in the LBDs upon neurophysiological biomarkers should be examined and reported in studies. This is necessary as it is possible that the use of common treatments such as cholinesterase inhibitors, psychotropics (e.g. antipsychotics, benzodiazepines, etc.) or anti-parkinsonian medications, might differentially affect EEG, MEG or TMS measures in particular. Subsequently, this is likely to impact upon the utility of the neurophysiological marker, as either a diagnostic tool, or as a marker of treatment efficacy.
(3) Efforts should be focussed on developing the efficacy of EEG as a diagnostic tool in LBD. Pragmatically, EEG is a relatively inexpensive technique, and the development of new methodologies and automated analysis techniques now mean that EEG can have greater diagnostic specificity compared to subjective visual or manual approaches (Bonanni et al., 2010, Snaedal et al., 2012). However, a standardised analytical approach to EEG, which can be applied in clinical situations, is still needed for EEG to be an effective neurophysiological diagnostic biomarker in LBD.
(4) Neurophysiological biomarkers are not usually examined in isolation, and combinative approaches with other biomarkers (neurophysiological or otherwise) might improve sensitivity and specificity (Shaw et al., 2007). For example, Hari and colleagues note that the combined use of MEG, EEG and structural and functional magnetic resonance imaging recordings provide the most information regarding brain function (Hari et al., 2010). In this context, for example, studies which could combine SAI or EEG with neuroimaging may improve their diagnostic utility, by examining both structural and functional divergences, and phenotypic variations between diseases such as DLB and AD.

## Figures and Tables

**Table 1:** Potential neurophysiological biomarkers in the Lewy body dementias.

Neurophysiological biomarker	Presumed functional significance in LBD	Example references
Autonomic measures: (cardiovascular function, heart rate variability, sympathetic sweat response, skin vasomotor reflex and sympathetic skin response)	Autonomic dysfunction is common and often an early symptom of LBD. Therefore, measures which detect autonomic system dysfunction might aid the early detection of LBD	Akaogi et al. (2009) and Negami et al. (2013)
Blink reflex	As brainstem neuropathology has been proposed to be a feature of early LBD, an abnormal blink reflex may indicate the development of LBD	Anzellotti et al. (2008) and Bonanni et al. (2007)
EEG: resting-state/coherence analysis	Resting-state EEG analytic methods (e.g. through the comparison of power spectra between groups) can assess underlying cortical activity, potentially providing an insight into neurotransmitter (e.g. cholinergic) deficits in LBD. Coherence analysis may allow the assessment of functional connectivity in the LBDs	
EEG: event-related potentials (visual or auditory)	Task-dependent event-related potential studies may allow the investigation of attentional and cognitive deficits in LBD	Kurita et al., 2010, Perriol et al., 2005 and Pugnetti et al. (2010)
MEG	MEG measures the same source signal as EEG, can measure oscillatory cortical function, and might be a measure of cholinergic deficits in LBD	Bosboom et al. (2009) and Franciotti et al. (2006)
TMS (SAI)	SAI is a tool which can be used to examine the cholinergic deficit in LBD	Celebi et al. (2012) and Di Lazzaro et al. (2007)

*Abbreviations:* EEG: electroencephalography; LBD: Lewy body dementia; MEG: magnetoencephalography; SAI: short afferent inhibition; TMS: transcranial magnetic stimulation.

## Appendix A Supplementary data

Supplementary Table S1 EEG studies.

Supplementary Table S2 Other Neurophysiological Techniques.
