# Supplementary material for: Neurophysiological biomarkers for Lewy body dementias
Source: Clin Neurophysiol. 2016 Jan;127(1):349–59. doi: 10.1016/j.clinph.2015.06.020 (PMC4727506; doi:10.1016/j.clinph.2015.06.020)
Supplement: Supplementary Table S1 — EEG studies. [file mmc1.docx]

| **Supplementary Table S1.**  *EEG studies* | | |  |
| --- | --- | --- | --- |
| Study | Participants | Findings | Medication details |
| Andersson *et al.* (2008) | DLB (*n* = 20), AD (*n* = 64), controls (*n* = 54). | Resting EEG. DLB patients showed greater delta-band power variability over 2 second intervals in parietal areas. DLB group had higher degree of overall delta coherence and a lower degree of overall alpha coherence than other groups. EEG measures (which) were able to distinguish DLB patients from AD patients and controls with area under the ROC curves ranging from 0.75 and 0.80 and between 0.91 and 0.97. | Antidepressants (DLB: 50%, AD: 39%, controls: 6%); benzodiazepines (DLB: 0%, AD: 19%, controls: 2%); neuroleptics (DLB: 50%, AD: 14%, controls: 0%) |
| Barber *et al*. (2000) | DLB (*n* = 18), probable AD (*n* =20). | Resting EEG assessed in both groups. EEG slowing in both groups (4-7Hz range) but no significant differences in EEG. Negative correlation between the EEG grade (calculated on basis of EEG features) and MMSE scores (*rs* = -0.61), indicating that a lower MMSE score was associated with more impaired EEG activity. | No information available. |
| Bonanni *et al*. (2008) | DLB (*n* = 50), AD (*n* = 50), PDD (*n* = 40). | Resting EEG. EEG variability assessed using mean frequency analysis and compressed spectral arrays. Differential EEG activity between DLB and AD groups in posterior derivations, with most DLB patients displaying a frequency band of 5.6-7.9Hz. Dominant frequency variability differed between AD and DLB patients. Only AD patients showed a pattern with dominant alpha bands. In DLB and PDD, residual alpha and 5.6-7.9Hz bands were related to presence and severity of cognitive fluctuations. | Patients were free from antidepressant, anticonvulsant, benzodiazepine, typical/atypical antipsychotic or anticholinergic medication. |
| Bonanni *et al*. (2010) | DLB (*n* = 36), AD (*n* = 40), controls (*n* = 50). | Auditory P300 responses recorded by an oddball paradigm. DLB patients showed a delayed P300, of lower amplitude, compared to AD patients. DLB patients also showed reversed anterior-to-posterior scalp amplitude gradient. The P300 latency showed positive correlations with NPI, ADAS-cog and CAF scores, and negative correlations with MMSE, FAB and DRS-2 scores. Gradient inversion and delayed frontal P300 responses were able to differentiate between DLB and AD with a sensitivity of 70% and specificity of 97%. | No information available. |
| Bonanni *et al*. (2015) | MCI (*n* = 47), with DLB (*n* = 50), AD (*n* = 50) and controls (*n* = 50). | Two-part study: cross-sectional study, where clinical, neuropsychological and EEG assessments were analysed at study admission, and prospective longitudinal study, where all groups were followed-up for three years, where clinical and neuropsychological assessments were repeated every six months. Of the individuals with MCI who converted to probable DLB (*n* = 20), 100% displayed EEG abnormalities typical of DLB (a dominant frequency of less than 8Hz and a dominant frequency variability of more than 1.5Hz). These EEG features were positively related to cognitive fluctuations. | No information available. |
| Briel *et al.* (1999) | DLB (*n* = 14), AD (*n* = 11). | Clinical EEG records were examined and rated (blind to diagnosis). DLB patients displayed a loss of dominant alpha activity and greater temporal lobe slow-wave transient activity compared to AD patients. | No information available. |
| Brønnick *et al*. (2010) | DLB (*n* = 17), PD (*n* = 16), PDD (*n* = 15), AD (*n* = 16), controls (*n* = 18). | ERP study using an auditory oddball-distractor task and mismatch negativity paradigm. PDD patients showed significantly less negative mismatch negativity than DLB patients, and smaller amplitudes than controls and PD groups. | Cholinesterase inhibitors (DLB: 53%, PDD: 60%, PD: 0%, AD: 56%, controls: 0%); L-dopa (DLB: 47%, PDD: 100%, PD: 69%, AD: 0%, controls: 0%); dopamine agonists (DLB: 0%, PDD: 20%, PD: 38%, AD: 0%, controls, 0%); antidepressants (DLB: 12%, PDD: 33%, PD: 31%, AD: 31%, controls: 0%); antipsychotics (DLB: 18%, PDD: 60%, PD: 25%, AD: 19%, controls: 0%) |
| Calzetti *et al*. (2002) | DLB (*n* = 10), AD (*n* = 9). | Resting EEG. Slowing of background activity (6-8Hz range) in all DLB patients and 6 of the 9 AD patients. FIRDA was present in 7 of the DLB patients. | No information available. |
| Caviness *et al*., (2007) | PD (*n* = 42), PD-MCI (*n* = 16); PDD (*n* = 8). | Resting EEG. PDD patients displayed lowest DPBRF compared to PD-MCI and PD patient groups. Significant differences also shown in delta between PDD and PD-MCI and in both delta and theta bands compared to PDD (percentage of global relative power increased in both bands). Dominant posterior background rhythm frequency and global relative power correlated with MMSE scores. | Participants were not taking benzodiazepine, anti-epileptic or antipsychotic medication. |
| Engedal *et al*. (2015) | Patients with depression (*n* = 12), subjective memory complaints (*n* = 63), MCI (*n* = 121), AD (*n* = 135), VaD (*n* = 15), DLB (*n* = 10), PDD (*n* = 5), other dementias (*n* = 4). | SPR was used on a database of resting-state EEG recordings. Quantitative EEG was able to separate AD patients from healthy individuals (sensitivity of 84% and specificity of 81%) snd LBD from AD with a sensitivity of 85% and specificity of 87%. | Antipsychotics (2%); antidepressants (19%); tranquilizer/hypnotic medication (7%); anti-dementia drugs (memantine and cholinesterase inhibitors; 6%); painkillers (5%) |
| Fernandez-Torre *et al.* (2007) | Single case study (68-year-old male DLB). | Retrospective analysis of 12 EEG recordings from same patient. EEGs during initial phase of disease showed a slowing of background activity: diffuse theta rhythms and occasional bilateral arrhythmic slow waves, with sporadic frontal transients. Moderate slowing of background activity observed during intermediate phase of disease with diffuse theta rhythms and frontal and fronto-central frequent multifocal waves, occasionally accompanied by right temporal spikes. EEGs during terminal phase indicated dominance of generalised periodic sharp wave complexes. | No information available. |
| Gu *et al.* (in press) | PD-MCI (*n* =17), PDD (*n* = 9) | Resting EEG. All patients were followed-up at 1-year and 2-year time points. PDD patients showed significantly increased beta peak frequency and decreased alpha relative power and alpha/theta power compared to PD-MCI and 1-year and 2-year time points. | No information available. |
| Helkala *et al.* (1991) | AD (*n* = 19), PDD (*n* = 18), controls (*n* = 14). | Resting EEG. Relationship between visual function, praxic functions, speech understanding, automatic speech, category fluency and slow-wave activity, explaining variance in AD group, but not in PDD or controls. Relationship between alpha activity and visual function, praxic function and list learning was observed in PDD group. | AD group: no centrally-active medication (*n* = 18), benzodiazepines (*n* = 1); PDD group: anticholinergic medication (*n* = 4), neuroleptics (*n* = 4), antidepressants (*n* = 4), levodopa (*n* = 15), seleginil (*n* = 6), bromocriptin (*n* = 6), amantadine (*n* = 1) |
| Kai *et al*. (2005) | DLB (*n* = 15), AD (*n* = 15), controls (*n* = 15). | Resting EEG. DLB patients showed higher delta and theta band coherence in frontotemporal regions compared to AD patients and controls. | Levodopa (DLB: *n* = 9, AD: *n* = 0); amantadine HCI (DLB: *n* = 4, AD: *n* = 0); dopamine agonist (DLB: *n* = 1, AD: *n* = 0). |
| Kurita *et al*. (2010) | PDD hallucinators (*n* = 11), PDD non-hallucinators (*n* = 6), DLB (*n* = 24), AD (*n* = 21). | EEG recorded to obtain visual and auditory ERPs. Facial discrimination paradigm (to elicit visual ERPs) and odd-ball paradigm (to elicit auditory ERPs). DLB patients and PDD patients with hallucinations exhibited greater visual P3 latencies (relative to AD). Compared to controls, the visual P2 latencies were greater in both PDD and DLB patient groups. | No information available, however patients taking antipsychotic medications were excluded from the study. |
| Lee *et al.* (in press) | DLB (*n* = 29), AD (*n* =54) | GTE scoring used to rate resting-state EEG recordings. Cut-off criteria was able to separate DLB from AD with sensitivity of 79% and specificity of 76%. | Cholinesterase inhibitors (DLB: *n* = 1, AD: *n* = 2); benzodiazepines (DLB: *n* = 3, AD: *n* = 1); antipsychotics (DLB: *n* = 3, AD: *n* = 0); antidepressants (DLB: *n* = 3, AD: *n* = 3); |
| Liedorp *et al*. (2009) | AD (*n* = 382), individuals with subjective memory complaints (*n* = 274), MCI (*n* = 190), individuals with an unspecified psychiatric disorder (*n* = 118), patients with frontotemporal lobar degeneration (*n* = 61), VaD (*n* = 53), DLB (*n* = 38). | Resting EEG recording. DLB, VaD and AD patients tended to exhibit focal and diffuse EEG abnormalities. AD patients tended to show diffuse abnormalities only. | No information available. |
| Londos *et al*. (2003) | DLB (*n* = 34), AD (*n* = 28). | EEG recordings were retrospectively analysed. No differences between DLB and AD groups were observed. | No information available. |
| Perriol *et al*. (2005) | DLB (*n* = 10), AD (*n* = 10), PDD (*n* = 10), controls (*n* = 10). | EEG was recorded during an auditory pre-pulse inhibition paradigm. Pre-pulse inhibition was reduced in the DLB group (relative to AD patients and controls). | No information available, |
| Pugnetti *et al.* (2010) | PD (*n* = 21), PDD (*n =* 7), LBD (*n* = 10), controls (*n* = 14). | Visual ERPs. EEG recorded during eyes opening and photic stimulation (12Hz). During photic stimulation posterior electrode activity was reduced in LBD and PD patients relative to controls (there was no reactivity in PDD patients). | No information available, |
| Roks *et al*. (2008) | DLB (*n* = 18), AD (*n* = 35), patients with subjective memory complaints (*n* = 36). | FRIDA found in 33.3% of the DLB and 2.9% of the AD patients. GTE scores could differentiate DLB and AD patients with sensitivity of 72% and a specificity of 85%. | Antipsychotic medication (DLB: *n* = 5; AD: *n* = 2; patients with subjective memory complaints: *n* = 0); cholinesterase inhibitors (DLB: *n* = 2; AD: *n* = 2; patients with subjective memory complaints: *n* = 1); benzodiazepines (DLB: *n* = 2; AD: *n* = 2; patients with subjective memory complaints: *n* = 7). |
| Schlede *et al*. (2011) | PD (*n* = 4), PD-MCI (*n* = 10), PDD (*n* = 5). | Resting EEG. Two EEG measures (short GTE score; Frequency of Rhythmic Background Activity) were negatively associated with a combined MMSE/CDT score. | No information available however patients taking neuroleptic drugs were excluded. |
| Snaedal *et al.* (2012) | LBD (combined DLB and PDD; *n* = 52), AD (*n* = 239), controls (*n* = 226), VaD (*n* = 58), MCI which was stable for >24 months (*n* = 41), FLD (*n* = 14), and depressed individuals (*n* = 24). | SPR was used on a database of resting-state EEG recordings, where classifiers, created for each possible pairs of groups, were based on 20 EEG spectral features and 37 EEG coherence features. The level of separation observed using SPR was good-to-excellent, but was reduced in situations with a high level of co-morbidity. | No information available. |
| Walker *et al*. (2000a) | DLB (*n* = 15), AD (*n* = 15), controls (*n* = 10). | Resting EEG (across 90 seconds) DLB patients had more severe fluctuating cognition (clinically assessed), more variability in objective attentional measures, and greater fluctuations in EEG frequencies than AD patients or controls. | No information available. |
| *Abbreviations:* AD: Alzheimer’s disease; ADAS-cog: Alzheimer’s disease Assessment Scale – cognitive; CAF: Clinician Assessment of Fluctuation; CDT: Clock Drawing Test; DLB: dementia with Lewy bodies; DPBRF: dominant posterior background rhythm frequency; DRS-2: Dementia Rating Scale-2; EEG: electroencephalography; ERP: event-related potential; FAB: Frontal Assessment Battery; FIRDA: Frontal intermittent rhythmic delta activity; FLD: frontal lobe dementia; GTE: Grand Total EEG; Hz: hertz; LBD: Lewy body dementias; MCI: mild cognitive impairment; MMSE: Mini-mental state examination; NPI: Neuropsychiatric Inventory; PD: Parkinson’s disease; PDD: Parkinson’s disease with dementia; SPR: Statistical pattern recognition; VaD: Vascular dementia. | | |  |
