# Supplementary material for: Neurophysiological biomarkers for Lewy body dementias
Source: Clin Neurophysiol. 2016 Jan;127(1):349–59. doi: 10.1016/j.clinph.2015.06.020 (PMC4727506; doi:10.1016/j.clinph.2015.06.020)
Supplement: Supplementary Table S2 — Other Neurophysiological Techniques. [file mmc2.docx]

| **Supplementary Table S2.**  *Other Neurophysiological Techniques* | | | | |  |
| --- | --- | --- | --- | --- | --- |
| Study | Technique | Participants | Details | Findings | Medication details |
| Akaogi *et al.* (2009) | Sympathetic sweat response, skin vasomotor reflex and cardiovascular function | DLB (*n* = 12), PDD (*n* = 12), PD (*n* = 12), healthy controls (*n* = 12). | Sympathetic sweat response and skin vasomotor reflex were measured from palm, and cardiovascular function, assessed using coefficient of variation of R-R intervals (CV_R-R­_), was measured in response to head-up tilt test. | DLB, PDD and PD patients showed reduced sweat response amplitudes compared to controls. Vasomotor reflex amplitudes were lower in DLB and PDD patients compared to controls. DLB also showed a lower CV_R-R­_ value than controls. | DLB: levodopa with decarboxylase inhibitor (*n* = 5); levodopa with decarboxylase inhibitor in combination with dopamine agonist (*n* = 3). PDD: levodopa with decarboxylase inhibitor in combination with dopamine agonist (*n* = 10), PD: levodopa (*n* = 1), levodopa with decarboxylase inhibitor (*n* = 2), cabergoline (*n* = 1), levodopa with decarboxylase inhibitor in combination with dopamine agonist (*n* = 4), selegiline (*n* = 2), amantadine hydrochloride (*n* = 1), droxidopa (*n* = 2). |
| Anzellotti *et al*. (2008) | Blink reflex | DLB (*n* = 26), AD (*n* = 20), healthy controls (*n* = 30). | Blink reflex evaluated at baseline, 1 week after administration of vitamin E, and 1 and 2 weeks after donezpil administration. R1, R2 and R3 evaluated. | R2 latency significantly decreased in DLB patients. | Patients participated prior to pharmacological treatment and were free from antidepressant, anticonvulsant, anticholinergic, atypical or typical antipsychotic medication, or cholinesterase |
| Bonanni *et al*. (2007) | Blink reflex | DLB (*n* = 26), multiple system atrophy (*n* = 26), PD (*n* = 26), AD (*n* = 20), controls (*n* = 30). | Blink reflex assessed by recording ipsilateral R1 and ipsilateral and contralateral R2 and R3. | R2 latency significantly increased in DLB, irrespective of the presence of RBD. | Patients who were taking antidepressants,  anticonvulsives, anticholinergics, typical or atypical  antipsychotics or cholinesterase inhibitors, were not  admitted to the study. |
| Bosboom *et al*. (2009) | MEG | PDD (*n* = 8). | Resting MEG before and after cholinesterase inhibitor treatment with rivastigmine | Rivastigmine treatment reversed some of the slowing of resting state activity resulting in an increase in power in the alpha range in parieto-occipital and temporal region, an increase in beta power (diffuse) and a decrease of delta power in fronto-central and parieto-occipital regions. | Combination of levodopa and decarboxylase inhibitor (*n* = 8); additional dopamine agonist (*n* = 6). |
| Celebi *et al*. (2012) | TMS (SAI) | **PD (*n* = 10), PDD (*n* = 10), AD (*n* = 10).** | SAI tested by holding TMS coil over contralateral FDI muscle in order to evoke MEP. | Impaired SAI (higher MEP) in AD and PDD compared to controls, with no differences between AD and PDD groups. There was a significant negative relationship between MEPs and MMSE scores irrespective of group. | Participants were free from cholinergic or anticholinergic medications in the two weeks prior to study participation. |
| Di Lazzaro *et al*. (2007) | TMS (SAI) | **DLB (*n* = 10); AD (*n* = 10); controls (*n* = 10).** | SAI tested by holding TMS coil over contralateral FDI muscle in order to evoke MEP.  contralateral FDI | SAI was significantly reduced in both DLB and AD patients relative to healthy controls. | No patients had been treated with cholinesterase inhibitors prior to study participation. |
| Franciotti *et al*. (2006) | MEG | AD (*n* = 15), LBD (*n* = 7), controls (*n* = 9). | MEG at rest (eyes open and closed) and during an acoustic oddball stimulation task. | Patients showed a significant reduction in alpha coherence relative to controls during resting conditions. Alpha near and far coherence in AD patients differed from controls in the left hemisphere. In LBD patients relative to controls a reduced alpha coherence was apparent in right near and far areas. | No participants had received antidepressant, anticonvulsivant, anticholinergic, l-Dopa, dopamine agonists, typical or  atypical antipsychotic agents or CNS-active drugs at the time of participation. |
| Kofler *et al*. (2001) | ASR | DLB (*n* = 8), PD (*n* = 10), MSA (*n* = 7), PSP (*n* = 10), healthy controls (*n* = 10). | Auditory tone bursts of a random frequency and intensity were presented to participants. EMG recordings obtained from a range of areas were used to measure the ASR. | DLB patients showed abnormal ASR profile: fewer ASRs and very few responses in lower extremities. DLB patients showed a similar probability of facial beck and upper extremity responses to PD or MSA patients. DLB patients also showed lower ASR amplitudes compared to PD patients or controls. | No information available. |
| Marra *et al.* (2012) | TMS (SAI) | DLB (*n* = 18), AD (*n* = 18) and separate data obtained from controls (*n* = 10). | SAI tested by holding TMS coil over contralateral FDI muscle in order to evoke MEP. | SAI reduced in AD and DLB compared to controls. SAI also positively correlated with overall NPI hallucinations subscale in DLB patients. | Participants were not being treated with cholinesterase inhibitors, antidepressant, or antipsychotic drugs at the time of participation. |
| Nardone *et al*. (2006) | TMS (SAI; SICI; MT; CMCT; ICF) | DLB (*n* = 10), AD (*n* = 13) controls (*n* = 15). | SAI tested by holding TMS coil over contralateral FDI muscle in order to evoke MEP. SICI and ICF also assessed using TMS over motor cortex. | Relative to healthy controls, SAI and SICI were significantly reduced in AD but not DLB patients. No significant differences between groups in ICF, MT or CMCT. | Participants were free from (unspecified) drugs which could potentially affect motor cortex excitability in the two weeks prior to participation. |
| Negami *et al.* (2013) | SSR & HRV | DLB (*n* = 20), AD (*n* = 20). | SSR measured in response to median nerve stimulation. HRV assessed whilst participants were seated. | SSR was lower in DLB patients compared to AD (sensitivity: 85% and specificity: 85%). HRV (ratio of low-frequency to high-frequency power) lower in DLB patients compared to AD (sensitivity: 90% and specificity: 85%). | No information available. |
| Taylor *et al*. (2011) | TMS | DLB (*n* = 21), age-matched controls (*n* = 19). | TMS used to investigate visual cortical hyperexcitability. TMS was used to elicit phosphenes in participants, which are considered to be a marker of visual cortex excitability. | Phosphene thresholds were not significantly different between groups. DLB patients showed a negative association between phosphene threshold and hallucinations severity and frequency (assessed using the NPI). | DLB: cholinesterase inhibitors (*n* = 16); anti-parkinsonian medication (*n* = 6) |
| *Abbreviations*:  ASR: auditory startle response; CMCT: central motor conduction time; CV_R-R­_: coefficient of variation of R-R intervals; DLB: dementia with Lewy bodies; EMG: electromyography; FDI: first dorsal interosseous; HRV: heart rate variability; ICF: intracortical facilitation; MEG: magnetoencephalography; MEP: motor-evoked potentials; MoCA: Montreal Cognitive Assessment; MT: motor threshold; MSA: multiple system atrophy; NPI: Neuropsychiatric Inventory; PD: Parkinson’s disease; PDD: Parkinson’s disease with dementia; PD-MCI: Parkinson’s disease with mild cognitive impairment; PSP: progressive supranuclear palsy; SAI: short afferent inhibition; SICI: short latency intracortical inhibition; SSR: sympathetic skin response; TMS: transcranial magnetic stimulation. | | | | |  |
